# Lead‐Free Halide Double Perovskite Cs_2_AgBiBr_6_ with Decreased Band Gap

**DOI:** 10.1002/anie.202005568

**Published:** 2020-06-22

**Authors:** Fuxiang Ji, Johan Klarbring, Feng Wang, Weihua Ning, Linqin Wang, Chunyang Yin, José Silvestre Mendoza Figueroa, Christian Kolle Christensen, Martin Etter, Thomas Ederth, Licheng Sun, Sergei I. Simak, Igor A. Abrikosov, Feng Gao

**Affiliations:** ^1^ Department of Physics Chemistry, and Biology (IFM) Linköping University 58183 Linköping Sweden; ^2^ Department of Chemistry KTH Royal Institute of Technology 10044 Stockholm Sweden; ^3^ Deutsches Elektronen-Synchrotron (DESY) 22607 Hamburg Germany; ^4^ State Key Laboratory of Fine Chemicals Institute of Artificial Photosynthesis DUT-KTH Joint Education and Research Centre on Molecular Devices Dalian University of Technology 116024 Dalian China; ^5^ Materials Modeling and Development Laboratory National University of Science and Technology “MISIS” Leninskii pr 4 119049 Moscow Russia

**Keywords:** Ag–Bi disorder, band-gap engineering, crystal engineering, Cs_2_AgBiBr_6_, lead-free double perovskites

## Abstract

Environmentally friendly halide double perovskites with improved stability are regarded as a promising alternative to lead halide perovskites. The benchmark double perovskite, Cs_2_AgBiBr_6_, shows attractive optical and electronic features, making it promising for high‐efficiency optoelectronic devices. However, the large band gap limits its further applications, especially for photovoltaics. Herein, we develop a novel crystal‐engineering strategy to significantly decrease the band gap by approximately 0.26 eV, reaching the smallest reported band gap of 1.72 eV for Cs_2_AgBiBr_6_ under ambient conditions. The band‐gap narrowing is confirmed by both absorption and photoluminescence measurements. Our first‐principles calculations indicate that enhanced Ag–Bi disorder has a large impact on the band structure and decreases the band gap, providing a possible explanation of the observed band‐gap narrowing effect. This work provides new insights for achieving lead‐free double perovskites with suitable band gaps for optoelectronic applications.

## Graphical abstract

**Disorderly conduct**: A crystal‐engineering strategy has been introduced to narrow the band gap of benchmark double perovskite Cs_2_AgBiBr_6_. The band gap of Cs_2_AgBiBr_6_ crystals can be reduced from 1.98 eV to 1.72 eV, reaching the smallest reported band gap for Cs_2_AgBiBr_6_ under ambient conditions. DFT calculations indicate that Ag–Bi disorder in the crystal structure could lead to band‐gap narrowing.

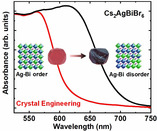

Lead (Pb) halide perovskites have attracted intensive attention because of their excellent optoelectronic properties, such as suitable band gaps, long hole‐electron diffusion length, and high defect tolerance.^1^, ^2^, ^3^, ^4^ However, the toxicity of Pb and poor stability represent the main bottlenecks for further commercial applications of these emerging materials. A wide range of different strategies have been employed to develop suitable Pb‐free alternatives.^5^, ^6^, ^7^, ^8^, ^9^ Among others, one of the most promising approaches is to replace the toxic divalent Pb^2+^ cation with a combination of monovalent and trivalent cations, forming double perovskites.^10^, ^11^, ^12^, ^13^ Lead‐free double perovskites (e.g. Cs_2_AgBiBr_6_) show attractive optical and electronic features.^13^, ^14^ However, the large band gap in Cs_2_AgBiBr_6_ is a main reason limiting their photovoltaic performance. To improve their device performance, finding effective strategies to narrow the band gap of the benchmark lead‐free double perovskite Cs_2_AgBiBr_6_ is highly desirable.

Along this direction, there have been several structural modification attempts, aiming to decrease the band gap of Cs_2_AgBiBr_6_. For example, Sb impurity doping has been successfully utilized to decrease the band gaps from 2.12 eV to 1.86 eV. The band gap reduction is due to the higher energy levels of Sb 5s states than those of Bi 6s states, raising the valence band maximum slightly.^15^ In addition, the phase transition induced by high pressure (15 GPa) leads to the band gap narrowing from original 2.19 eV to 1.70 eV in Cs_2_AgBiBr_6_ crystals.^16^ Very recently, a temperature‐induced structural change was demonstrated in both Cs_2_AgBiBr_6_ crystals and thin films, resulting in band gap decrease behavior (thermochromism) at high temperatures.^14^ Unfortunately, these “low‐band gap” phases in the previous reports either still have significantly too large band gaps or are unstable at ambient conditions, limiting their device applications.

Herein, we employ a crystal engineering strategy to modify the benchmark double perovskites Cs_2_AgBiBr_6_. Through simply controlling the crystal growth temperature and growth speed, we obtain modified Cs_2_AgBiBr_6_ crystals with a decreased band gap of 1.72 eV, which is the lowest value among all the reports for pure Cs_2_AgBiBr_6_ at ambient conditions. A 0.26 eV band gap narrowing is confirmed by comparing the absorption and emission spectra between modified and benchmark Cs_2_AgBiBr_6_ crystals. We use density functional theory (DFT) calculations to show that the presence of Ag–Bi disorder in Cs_2_AgBiBr_6_ introduces defect states in the gap and, as the disorder level increases, produces a band of defect states.

Cs_2_AgBiBr_6_ single crystals with different band gaps are synthesized by slow evaporation of the precursor solution at different temperatures. The key point to this method is to properly control the growth temperature and growth rate. Figure 1 a is a schematic illustration of the Cs_2_AgBiBr_6_ single crystal preparation. We optimize the growth conditions systematically, and achieve high‐quality Cs_2_AgBiBr_6_ crystals at evaporation temperatures of 60 °C (DP‐60) and 150 °C (DP‐150), respectively. Figure 1 b shows the optical images of DP‐60 and DP‐150, both of which crystalize in the octahedral shape. As the growth temperature increases from 60 °C to 150 °C, the colors of the resulting Cs_2_AgBiBr_6_ single crystals change from red to black, indicating a wider absorption range. In order to exclude the influence of surface effects, we crush a large black crystal into small pieces, and find that the cross‐sections of the small pieces are also black (Figure S1 in the Supporting Information). In addition, by extending the crystal growth time, we achieve a near centimeter‐sized single crystal (Figure S2), which is beneficial for single‐crystal devices.

**Figure 1:**
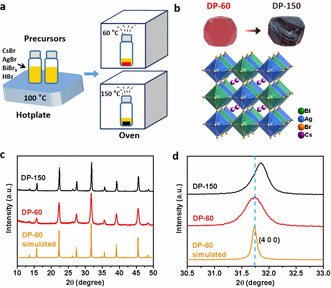
a) Procedure for the crystal growth of DP‐60 and DP‐150. b) Optical images and the crystal structure of the DP‐60 and DP‐150 single crystals. c) Normalized PXRD patterns of DP‐60 and DP‐150 crystals. d) The enlarged view of reflection (400) in the PXRD patterns. The simulated XRD of DP‐60 is obtained from Mercury software simulation.

We perform X‐ray diffraction (XRD) measurements, and exclude the possibility that the color change from DP‐60 to DP‐150 results from different crystal structures. Single crystal XRD (SCXRD) measurements (Table S1) indicate that both crystals show the typical double‐perovskite structure with alternating AgBr_6_ and BiBr_6_ octahedra (Figure 1 b). In the DP‐150 sample, we observe slight lattice shrink from 11.2695 Å to 11.2636 Å (Table S1), which is also consistent with the powder XRD (PXRD) measurements. The PXRD patterns of DP‐60 and DP‐150 are nearly identical, matching well with the simulated pattern (Figure 1 c), except for the (400) reflection which shifts slightly toward higher angle for DP‐150 (Figure 1 d), confirming slight lattice shrink. These XRD results are also consistent with energy dispersive spectroscopy (EDS) measurements, which indicate that both crystals have similar element distributions (Figure S3).

We quantify the absorption edges of DP‐60 and DP‐150 by measuring the UV/Vis diffuse reflectance spectra (Figure S4). The reflectance spectra are converted to pseudo‐absorbance spectra using the Kubelka‐Munk transform.^17^ As shown in Figure 2 a, the DP‐60 crystal shows a sharp absorption edge at approximately 625 nm, whereas the DP‐150 crystal shows an approximately 90 nm red‐shift with the absorption edge at around 715 nm. The corresponding band gap calculated from the Tauc plots is reduced from 1.98 eV for DP‐60 to 1.72 eV for DP‐150 (Figure 2 b), corresponding well with the color change of crystals (Figure 1 b).

**Figure 2:**
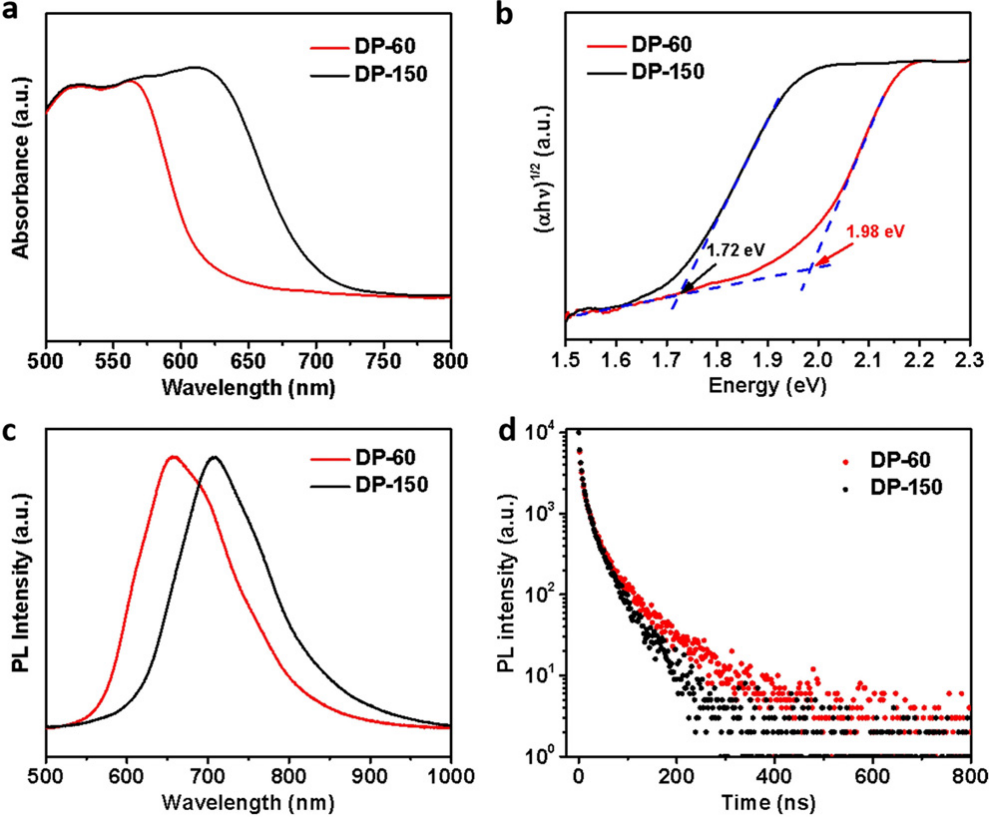
a) Normalized UV/Vis absorption spectra of DP‐60 and DP‐150 single crystals. b) Tauc plots of DP‐60 and DP‐150 for indirect band gap semiconductor,^9^ showing indirect band gaps of 1.98 eV for DP‐60 and 1.72 eV for DP‐150. Normalized PL spectra (c) and time‐resolved PL (d) of DP‐60 and DP‐150 single crystals at room temperature.

We then measure the photoluminescence (PL) spectra of both crystals, and find two interesting observations. Firstly, as shown in Figure 2 c, the PL spectrum of DP‐150 shows an obvious red shift compared with that of DP‐60. Such a red shift in the PL spectra is consistent with the red shift observed in the absorption measurements, confirming that the absorption edge shift results from the band gap narrowing effect (rather than simply the emergence of isolated defect states without changes to the bands).^18^ Secondly, the PL spectra of both crystals are relatively broad, and both of them can be fitted with two Gaussian peaks (Figure S5). A broad PL spectrum in Cs_2_AgBiBr_6_ crystals is consistent with previous reports,^9^, ^15^, ^19^ and two Gaussian peaks are ascribed to the emission from the band edge and defect states, respectively.^15^ To further understand the defect states, we perform the time‐resolved PL for DP‐60 and DP‐150 single crystals at room temperature, both of which exhibit a fast initial drop followed by a slower decay (Figure 2 d). The shorter PL decay time of DP‐150, combined with qualitatively weaker PL intensity (Figure S6), indicates more defect states in DP‐150.

We are now motivated to understand the origins of defect states and band gap narrowing in our crystals. We are tempted to consider the degree of Ag–Bi disorder as an important source of defect states and band gap narrowing, as different evaporation temperature, which is the only difference when preparing these two different crystals, makes it possible to arrange Ag–Bi atoms in different ways. We note that it is highly challenging to explicitly examine the Ag–Bi disorder in the crystal structure by present characterization techniques (Figure S7). On the other hand, density functional theory (DFT) is known as a reliable tool for investigations of materials properties. We therefore employ first‐principles DFT calculations to understand the effect of Ag‐Bi disorder on the electronic structure of Cs_2_AgBiBr_6_. We construct supercells with varying degrees of Ag–Bi disorder, ranging from the ideal rocksalt atomic arrangement to a structure representing the completely random 50%–50 % alloy on the Ag‐Bi sites (see Methods for details). These structures are labeled by a pair of numbers corresponding to the average numbers of nearest neighbors (NNs) of the opposite and the same kind. For example, the perfect rocksalt Ag–Bi ordering is denoted (6,0) since each Ag (Bi) ion has 6 Bi (Ag) and 0 Ag (Bi) NNs. The completely disordered case is hence labelled (3,3).

The (partial) electronic density of states (DOS) of this series of structures, obtained with the hybrid HSE06 functional including spin‐orbit coupling (HSE06+SOC), is shown in Figure 3. In Cs_2_AgBiBr_6_ the top of the valence band is made up primarily of hybridized Ag‐d/Br‐p states (with some Bi‐s character mixed in at the band edge), while the conduction band is made up of Bi‐p/Br‐p states. The (5.5,0.5) structure corresponds to one NN Ag‐Bi antisite pair in the supercell, that is, in the otherwise perfect rocksalt structure the positions of one Ag and one Bi are swapped. We see that this introduces an acceptor‐like defect state of Bi‐p/Br‐p character into the band gap. This type of defect state could be responsible for the double‐peak nature of the PL emission of DP‐60, discussed above. As the disorder is further increased, this defect state moves deeper into the gap and another, progressively broader set of defect states are formed below the conduction band edge. In addition, an occupied defect state of Ag‐d/Br‐p character emerges from the valence band edge into the gap (at *E*=0 in the (4.5,1.5) structure). Figure S8 displays effective band structures obtained with the PBEsol functional for the (5.5,0.5) and the (4.5,1.5) structures.

**Figure 3:**
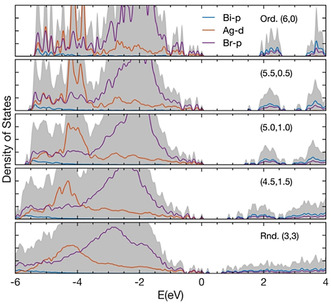
Calculated total and partial electronic density of states for a set of Cs_2_AgBiBr_6_ structures with varying degrees of Ag–Bi disorder (see text). The energy, *E*, is set to zero at the highest occupied state for each separate structure.

A quantitative statement regarding the actual level of the Ag–Bi disorder in our DP‐60 and DP‐150 crystals cannot be made with available data. However, the qualitative picture of a set of defect states that progressively emerges from the conduction band edge, and broadens, as the Ag–Bi disorder is increased (Figure 3), is consistent with the observed red‐shift of the absorption edge of DP‐150 in comparison to the DP‐60 being an effect of an increase in Ag–Bi disorder, and with the dual‐peak nature of the PL spectra of both crystals.

In addition, stability is an important issue to evaluate with regards to the potential use of perovskite materials for optoelectronic applications. We have therefore investigated the environmental stability of the DP‐150 Cs_2_AgBiBr_6_ double perovskites. The PXRD measurements (Figure 4 a) indicate that the freshly prepared DP‐150 crystals show no signs of decomposition for up to 240 days in the ambient atmosphere. In addition, this excellent stability of DP‐150 is also confirmed by complementary characterization techniques (Raman, absorption and PL spectra). The Raman band located at 178 cm^−1^ belongs to the symmetric stretching of Br atoms around Bi atoms in the octahedron,^20^ showing that no changes in the molecular bonds of the crystalline structure are present after a long time (Figure 4 b). Figure 4 c,d show the absorption and PL spectra of DP‐150, where negligible changes are observed after exposure to ambient atmosphere for 240 days, demonstrating excellent stability of DP‐150. Such a superior environmental stability makes DP‐150 a promising candidate for applications in highly stable single crystal optoelectronic devices.

**Figure 4:**
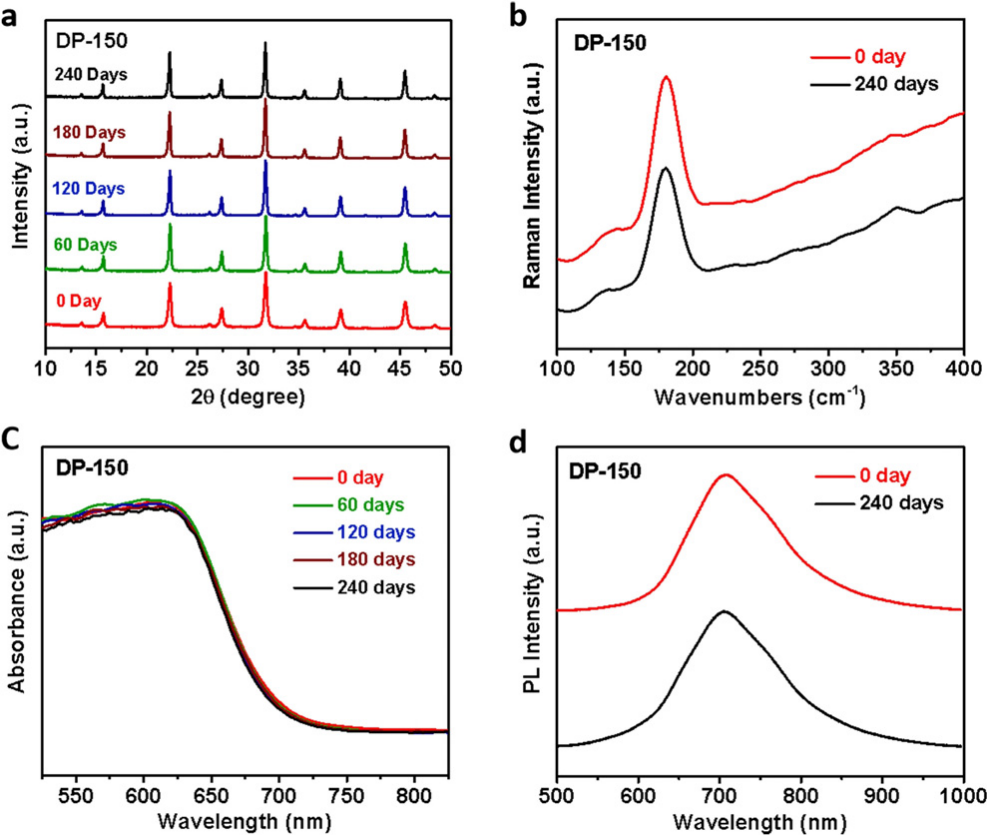
Time‐dependent PXRD (a), Raman spectra (b), UV/Vis absorption spectra (c) and PL spectra (d) of DP‐150 after exposure to ambient conditions.

In summary, we achieve the smallest reported band gap lead‐free double perovskite Cs_2_AgBiBr_6_ through controlling the growth temperature of single crystals. Although different growth temperatures have no effect on the crystal structures, the crystal prepared from high evaporation temperature (DP‐150) shows a significant band gap narrowing (ca. 0.26 eV) compared with that prepared from the low evaporation temperature (DP‐60). We hypothesize that this band gap narrowing is caused by an increased level of Ag–Bi disorder in DP‐150 as compared to DP‐60. First‐principles calculations show that when such disorder is introduced and progressively increased, it initially results in isolated defect states in the gap and eventually produces a band of defect states. We believe that the DP‐150 single crystals with decreased band gap could be suitable for high‐efficiency and stable single crystal devices, such as solar cells and photodetectors. In addition, our results provide a simple yet effective way to optimize the band gap of double perovskites and may be applicable to other crystalline materials as well.

## Conflict of interest

The authors declare no conflict of interest.

## Supporting information

As a service to our authors and readers, this journal provides supporting information supplied by the authors. Such materials are peer reviewed and may be re‐organized for online delivery, but are not copy‐edited or typeset. Technical support issues arising from supporting information (other than missing files) should be addressed to the authors.

Supplementary
