# Supplementary material for: Lead‐Free Halide Double Perovskite Cs2AgBiBr6 with Decreased Band Gap
Source: Angew Chem Int Ed Engl. 2020 Jun 22;59(35):15191–4. doi: 10.1002/anie.202005568 (PMC7496408; doi:10.1002/anie.202005568)
Supplement: Supplementary file 1 — Supplementary [file ANIE-59-15191-s001.pdf]

Angewandte Chemie
Eine Zeitschrift der Gesellschaft Deutscher Chemiker

Supporting Information

Lead-Free Halide Double Perovskite $Cs_2AgBiBr_6$ with Decreased Band Gap

Fuxiang Ji, Johan Klarbring, Feng Wang, Weihua Ning, Linqin Wang, Chunyang Yin, José Silvestre Mendoza Figueroa, Christian Kolle Christensen, Martin Etter, Thomas Ederth, Licheng Sun, Sergei I. Simak, Igor A. Abrikosov, and Feng Gao**

anie_202005568_sm_miscellaneous_information.pdf

Supporting Information

1. Experimental Section

Chemicals and Materials

All the chemicals used were purchased from Sigma-Aldrich without any further purification.

Preparation of $Cs_2AgBiBr_6$ crystals. Solid CsBr (213 mg, 1.0 mmol), $BiBr_3$ (224 mg, 0.5 mmol) and AgBr (94 mg, 0.5 mmol) were dissolved in 16 mL 48% HBr and then the mixture was transferred into an oven. The solvent was slowly evaporated at 60 ^oC and 150 ^oC, respectively, and crystals were obtained after the solvent was completely evaporated.

Physical measurements

The XRD patterns of the products were recorded with a X'Pert PRO X-ray diffractometer using Cu Kα1 irradiation (λ = 1.5406 Å). The Ultraviolet–visible absorption spectra were measured with a PerkinElmer model Lambda 900. Steady-state photoluminescence spectra were recorded with a 405 nm laser and an Andor spectrometer (Shamrock sr-303i-B, coupled to a Newton EMCCD detector). Energy dispersive X-ray spectroscopy (EDS) analysis was performed using a LEO 1550 SEM operated at 18 kV accelerating voltage, with an Oxford Instruments X-Max 80 mm^2 SDD detector. The time-correlated single-photon counting (TCSPC) measurements were performed on an Edinburgh Instruments spectrometer (FLS1000) with a 405 nm pulsed laser (<100 ps, 500kHz). The total instrument response function (IRF) was less than 30 ps. Raman spectra were recorded using a Raman spectrograph (Andor Kymera) connected to a Nikon Ti-E fluorescence microscope, using a 60x Nikon objective with 0.7 N.A. A laser with a nominal wavelength of 532 nm was used as an excitation source, the laser output power was 1 mW. Spectra were recorded with an acquisition time of 0.5 s. The signal was detected using a thermoelectrically cooled (-55 ^oC) Andor Newton EMCCD camera.

X-ray single crystallography

The single-crystal X-ray diffraction data for DP-60 and DP-150 were collected at 298 K by using Cu Kα radiation on a Bruker D8 VENTURE single crystal X-ray diffractometer (SCXRD) equipped with a kappa geometry goniometer. Data reductions and absorption corrections were performed with the APEX3 suite. Structures were solved by a direct method using the SHELXL-97 software package.[1] The crystal structure was refined using full-matrix

least-squares based on F^2 with all non-hydrogen atoms anisotropically defined. Hydrogen atoms were placed in calculated positions by means of the "riding" model. The details about data collection, structure refinement and crystallography are summarized in Table S1.

Optical band-gap determination

The reflectance spectra we obtained were converted to pseudo-absorbance spectra using the Kubelka-Munk transform.[2]

$$\alpha \approx \frac{(1-R)^2}{2R}$$

where α = pseudo-absorbance and R = reflection. The indirect bandgaps were measured by taking the intercept upon extrapolation of the liner regions of $(\alpha h\nu)^{1/2}$ Vs E(eV) plots.

High-energy pair distribution function (PDF)

Total scattering data were collected at the P02.1 beamline at PETRA III at DESY with a wavelength of 0.20697 Angstrom and a total exposure time of 10 min. All the data were collected in the same detector position; 281 mm sample to detector distance positioned with the beamspot centered at the detector to collect full Debye-Scherrer rings with maximum Q of 19.2 A^{-1}. Raw image processing into intensity vs. scattering angle was performed in the DAWN Science software package.[3] Transformation of scattering data to Pair Distribution Functions were carried out using the PDFgetX3 software package.[4] The rpoly value was set to 0.9, Q min was set to 0.1 A^{-1}, and Q max-inst was set to 19.2 A^{-1}.

Density Functional Theory calculations

Our DFT calculations were performed in the framework of the projector augmented wave (PAW)[5] method using the Vienna Ab Initio Simulation Package (VASP).[6,7] We treated exchange-correlation effects using the PBEsol[8] and the hybrid HSE06 functionals[9], as described in the main text. The Kohn-Sham orbitals were expanded in plane waves up to a kinetic energy cutoff of 400 eV. We used supercells containing 240 atoms, constructed from the primitive face-centered cubic (fcc) unit cell by a matrix M as $(\mathbf{A},\mathbf{B},\mathbf{C})^T = M(\mathbf{a},\mathbf{b},\mathbf{c})^T$, where $(\mathbf{A},\mathbf{B},\mathbf{C})$ and $(\mathbf{a},\mathbf{b},\mathbf{c})$ are the supercell and primitive cell lattice vectors, respectively and M is given by

$$\begin{pmatrix} 3 & 0 & -1 \\ 2 & -3 & 0 \\ 1 & 0 & -3 \end{pmatrix}.$$

This size of the supercell provides a fair tradeoff between computational complexity and ability to span varying degrees of Ag-Bi disorder within the cell. For each distribution of Ag and Bi all atomic positions were relaxed at the static equilibrium volume of the perfectly ordered cell (lattice constant, a = 11.18 Å), using the PBEsol functional without SOC, Γ-point sampling of the Brillouin zone (BZ) and a Gaussian smearing with a width of 0.05 eV, until the residual atomic forces were < 10^{-2} eV/Å. We note that $Cs_2AgBiBr_6$ in the cubic double perovskite structure is unstable towards phonon modes corresponding to tilting of $AgBr_6$/$BiBr_6$ octahedra.[10] Therefore, such atomic displacements will unavoidably be present in the relaxed disordered structures. Hence, to facilitate the comparison between the ordered and disordered structures, we relaxed also the perfectly ordered structure starting from slightly displaced Br atomic positions.

The electronic density of states (DOS) was then calculated for five different distributions (see below) using the HSE06 functional including spin-orbit coupling. The Γ-point BZ sampling and Gaussian smearing with a width of 0.05 eV were used. A denser k-point grid would be desirable but it is currently unaffordable with the available supercomputer power. We note that such single point BZ sampling may result in a DOS with somewhat overly localized peaks, but the qualitative physical picture is described correctly.

The supercell used to represent the completely random Ag-Bi distribution was generated using the special quasi-random structure (SQS)[11] technique. This entails distributing the Ag and Bi atoms in the limited sized supercell in such a way as to mimic a random alloy, by ensuring that the pair short-range order parameters (in our case up the 5th coordination shell on the Ag/Bi sublattice) are equal to zero. The partially disordered structures were generated to obtain certain target average Ag/Bi local environments. We label these structures by a pair of numbers (6-X,X), signifying that the Ag ions have, on average in the supercell, X nearest neighbor Ag atoms and 6-X nearest neighbor Bi atoms on the Ag/Bi sublattice. The perfectly ordered double perovskite structure thus corresponds to (6.0,0.0), i.e., all nearest neighbors to Ag are Bi, while the completely random distribution is (3.0,3.0). Note that disorder levels approaching perfectly random mixture is only likely to be observed at significantly higher temperatures than those considered here.[12] Still, including such structures into our simulations allows us to obtain a more complete picture of the influence of chemical disorder on the electronic structure of $Cs_2AgBiBr_6$.

In Figure S8 we show the effective band structures (EBSs) of the (4.5,1.5) and (5.5,0.5) structures using the PBEsol functional including SOC. These EBSs were obtained by

unfolding the band structure of the supercells to the primitive cell BZ using the methodology described by Popescu and Zunger[13,14], as implemented in the BandUP code.[15,16] In the case of perfect order, the EBS reduces, essentially, to the primitive cell band structure, and perturbations on the band structure due to the disorder can thus be gauged by comparing the EBS of a disordered structure to the primitive cell band structure. In the (5.5,0.5) case one clearly sees from the EBS that the main perturbations due to disorder on the primitive cell band structure is a splitting of the valence band edge around the X-point and a defect-like state below the conduction band edge. When the disorder is increased to the (4.5,1.5) case, the perturbations on the primitive band structure are much more significant. Note that these EBSs were obtained using PBEsol+SOC (to mitigate the overwhelming computational cost) and are thus not one-to-one comparable with the corresponding DOSs from the main text, which were calculated with HSE06+SOC.

2. Supporting Figures and Tables

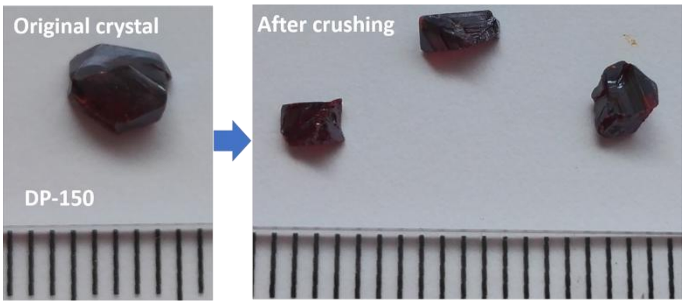

Figure S1. Optical images before and after crushing the DP-150 single crystal.

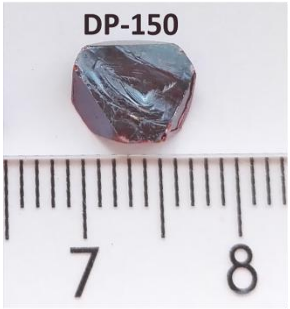

Figure S2. Optical image of centimeter-level DP-150 single crystal.

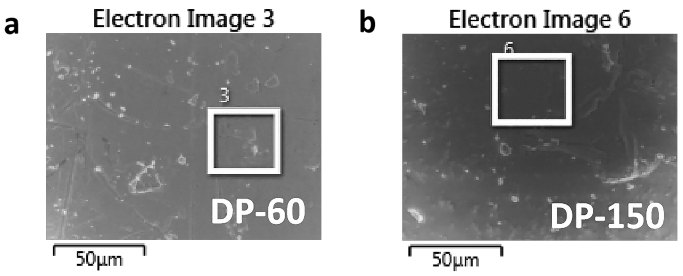

Element	Atomic %
Br	59.46
Ag	10.62
Cs	20.14
Bi	9.78
Total:	100.00

Element	Atomic %
Br	59.60
Ag	10.50
Cs	20.15
Bi	9.75
Total:	100.00

Figure S3. Energy dispersive spectroscopy (EDS) analysis of DP-60 and DP-150 single crystals.

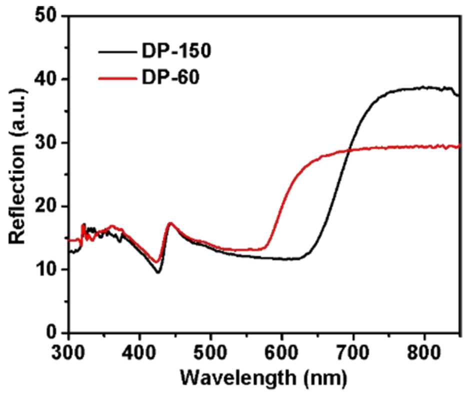

Figure S4. Reflectance spectra of DP-60 and DP-150 single crystals.

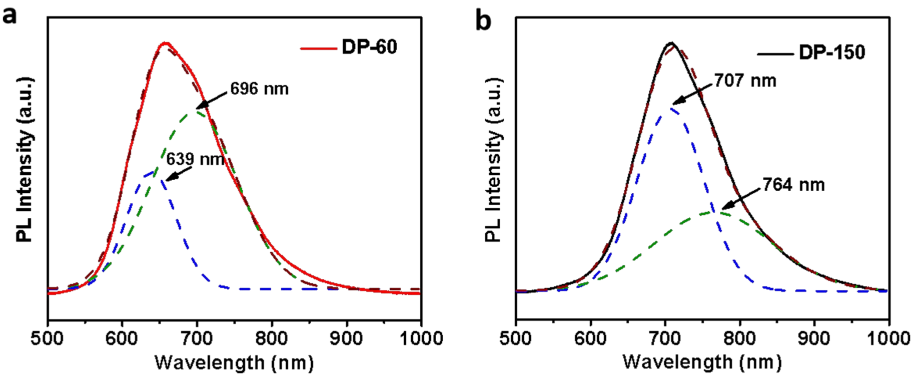

Figure S5. The individual PL spectra fitted using a Gaussian function for DP-60 (a) and DP-150 (b). The blue, green and brown dashed lines are fit peak 1, fit peak 2 and cumulative fit peak.

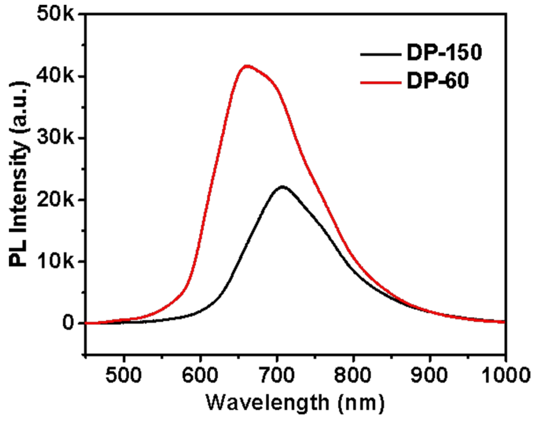

Figure S6. PL spectra of DP-60 and DP-150 single crystals.

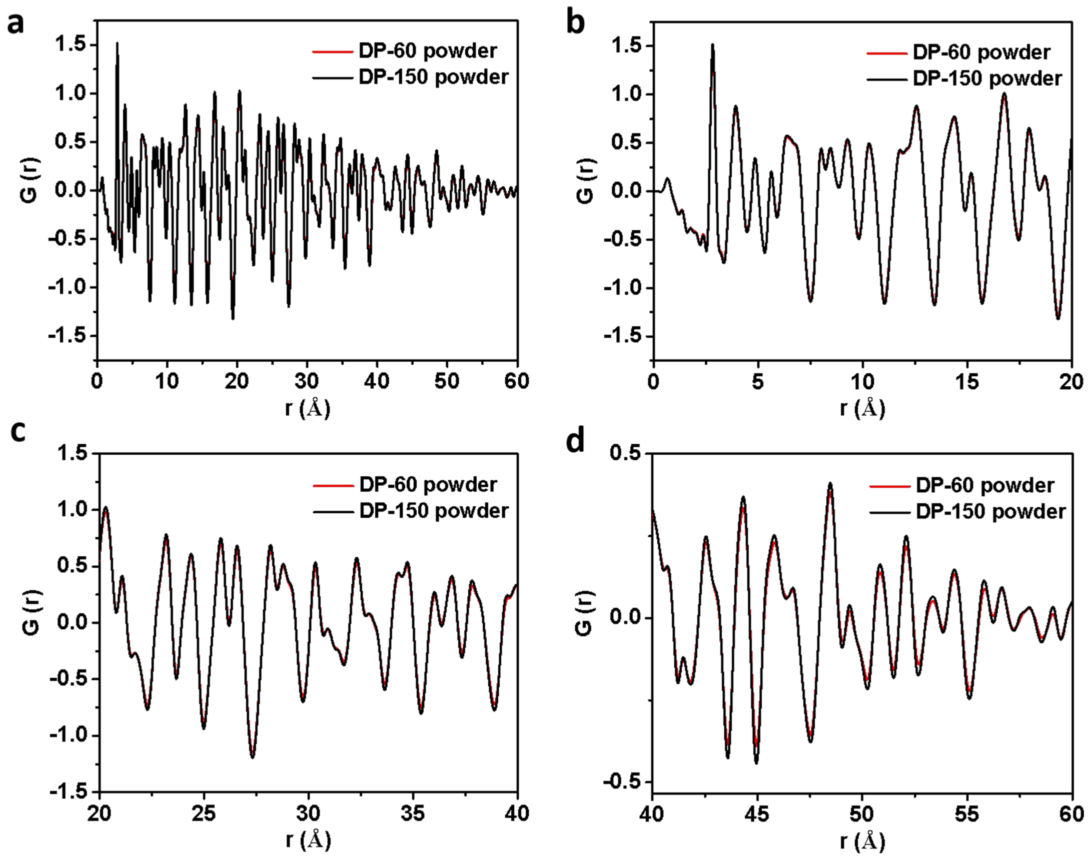

Figure S7. (a) High-energy pair distribution function (PDF) for DP-60 and DP-150. (b-d) Local regions of the PDFs. We observe negligible changes of the peak positions and intensities in the PDF. We note that the absence of difference in the PDF spectra does not mean that there is no difference in the degree of Ag-Bi disorder.[17] Instead, the Ag-Bi disorder is not visible in PDF measurements in this case.

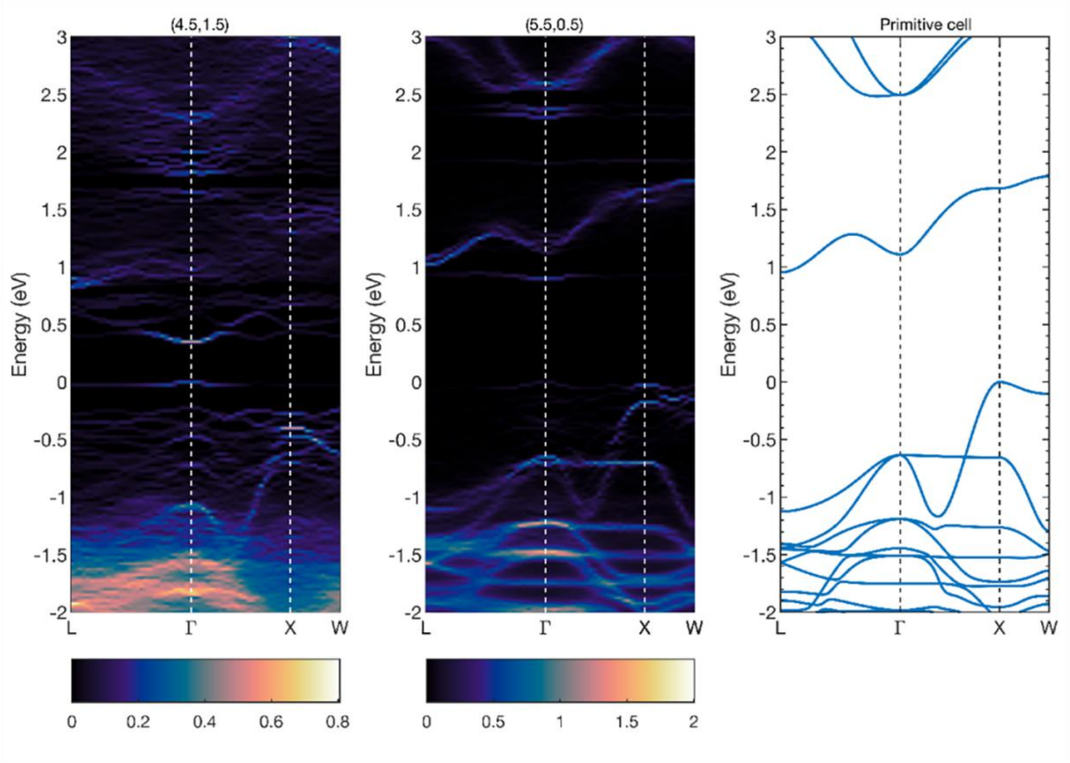

Figure S8. Band structure of the primitive cell (right) and effective band structures (EBS) for the (4.5,1.5) (left) and (5.5,0.5) (center) structures obtained with the PBEsol functional including SOC. Zero energy is aligned with the highest occupied state in all three cases. For display purposes all spectral weights (arb. units) above 70 % of the maximum are displayed with the same color.

Table S1: Crystallographic data and refinement parameters for double perovskites DP-60 and DP-150 at room temperature.

Compound	**DP-60**	**DP-150**
Temp. (K)	298(2)	298(2)
Wavelength (Å)	0.71073	0.71073
Formula	$Cs_2AgBiBr_6$	$Cs_2AgBiBr_6$
Formula weight	1062.13	1062.13
Space group	*F*m-3m	*F*m-3m
CCDC no.	1531091	1531091
Crystal system	Cubic	Cubic
a (Å)	11.2695(4)	11.2636(15)
b (Å)	11.2695(4)	11.2636(15)
c (Å)	11.2695(4)	11.2636(15)
α, β, γ (°)	90	90
V(Å^3)/Z	1431.24(9), 4	1429.0(3), 4
ρ (g·cm^{-1})	4.929	4.937
F(*000*)	1800	1800
Abs.coeff. (mm^{-1})	35.359	35.415
θ Range for data collection (°)	3.13 - 27.33	3.13 - 27.07
Index ranges	$-14 \leq h \leq 12$	$-14 \leq h \leq 14$
	$-11 \leq k \leq 14$	$-14 \leq k \leq 14$
	$-14 \leq l \leq 13$	$-14 \leq l \leq 14$
R_{int}	0.0576	0.1112
Independent reflect. /restraints /parameters	115 / 0 / 7	111 / 0 / 7
Refinement method	The least square refinement on F^2	
Goodness of fit on F^2	1.150	1.355
R_1, wR_2[a] [I>2σ(I)]	0.0325, 0.0637	0.0345, 0.0740
R_1, wR_2[a] [all data]	0.0331, 0.0639	0.0348, 0.0742
Residual (e·Å^{-3})	1.011 / -3.010	1.229 / -2.714

$R_1 = \sum||F_o| - |F_c||/|F_o|$, $wR_2 = [\sum w(\sum F_o^2 - F_c^2)^2/\sum w(F_o^2)^2]^{1/2}$

References

[1] G. M. Sheldrick, *SHELXL-97 Program Cryst. Struct. Refinement* **1997**, University of Göttingen: Göttingen, Germany.

[2] P. Kubelka, F. Munk, *Z Tech Phys* **1931**, *12*, 16.

[3] J. Filik, A. W. Ashton, P. C. Y. Chang, P. A. Chater, S. J. Day, M. Drakopoulos, M. W. Gerring, M. L. Hart, O. V. Magdysyuk, S. Michalik, et al., *J. Appl. Crystallogr.* **2017**, *50*, 959–966.

[4] P. Juhás, T. Davis, C. L. Farrow, S. J. L. Billinge, *J. Appl. Crystallogr*. **2013**, *46*, 560–566.

[5] P. E. Blöchl, *Phys. Rev. B* **1994**, *50*, 17953–17979.

[6] G. Kresse, D. Joubert, *Phys. Rev. B* **1999**, *59*, 1758–1775.

[7] G. Kresse, J. Furthmüller, *Phys. Rev. B* **1996**, *54*, 11169–11186.

[8] J. P. Perdew, A. Ruzsinszky, G. I. Csonka, O. A. Vydrov, G. E. Scuseria, L. A. Constantin, X. Zhou, K. Burke, *Phys. Rev. Lett.* **2008**, *100*, 136406.

[9] J. Heyd, G. E. Scuseria, M. Ernzerhof, *J. Chem. Phys.* **2003**, *118*, 8207–8215.

[10] Klarbring et al., *arXiv*:1912:05351, **2019**.

[11] A. Zunger, S.-H. Wei, L. G. Ferreira, J. E. Bernard, *Phys. Rev. Lett.* **1990**, *65*, 353–356.

[12] J. Yang, P. Zhang, S.-H. Wei, *J. Phys. Chem. Lett.* **2018**, *9*, 31–35.

[13] V. Popescu, A. Zunger, *Phys. Rev. B* **2012**, *85*, 085201.

[14] V. Popescu, A. Zunger, *Phys. Rev. Lett.* **2010**, *104*, 236403.

[15] P. V. C. Medeiros, S. Stafström, J. Björk, *Phys. Rev. B* **2014**, *89*, 041407.

[16] K. Lee, W. S. Yun, J. D. Lee, *Phys. Rev. B* **2015**, *91*, 125420.

[17] T. Egami, S. J. L. Billinge, Underneath the Bragg Peaks, *Mater. Today* **2003**, *6* (6), 57.
